# Allegheny County Medical Society

**Published:** 1891-11

**Authors:** 


### ALLEGHENY COUNTY MEDICAL SOCIETY.

### SCIENTIFIC MEETING, AUGUST 18, 1891.

T. D. Davis, M. D., President, in the chair.

A paper on "Suprapubic Cystotomy" was read by Dr. W. R. Stewart. See page 519.

Dr. Buchanan: I am very well acquainted with the history of the fourth case reported by Dr. Stewart, and I think the doctor deserves the greatest credit for the way in which he followed up the treatment. The case was a very mysterious one, and yet remains so. There was no obstruction of the urethra. There was no active cystitis. There was no disease in the kidney, or in the pelvis of the kidney, as far as could be discovered. There was nothing to give rise to the dilatation of the ureter, the enlarged outlet of which could be felt very plainly with the finger through the perineal incision and demonstrated with the sound, except the constant contraction of the bladder. The opening of the bladder for permanent drainage above the pubis was an entirely arbitrary matter, not based on anything except the fact that during the time at which the bladder was open below, the patient was relieved from pain. For this reason I think Dr. Stewart deserves the more credit for following up that hint and doing this operation without any other indication; an operation which has certainly proved very successful. The man was in a wretched condition; the contractions of the bladder were so painful as to make him cry out; he could not stand still when passing his water, and there was very extensive protrusion of the rectum.

Dr. Macfarlane: I have nothing to say except to compliment the doctor upon the manner of presenting his cases. There is one feature about the one case in which I cannot help but admire the manner in which he treated it. The case is the one in which he had rupture of the urethra. Now anybody who has ever attempted to do anything with rupture of the urethra knows the difficulty connected with it. I have on two occasions seen men of ample experience spend two hours or more before being able to unite the urethra ; on another occasion an hour and a half was spent with lack of success, the work being left to be completed at a later time, the man being, in the interval, in a precarious condition. Now, the doctor's method of treating that, I think, deserves widespread circulation, for it certainly acted very well indeed, and affords a very happy escape from the great difficulty connected with a case of rupture of the urethra.

DR. MCKENNAN : I have a specimen which may be of interest to the members of the society. It was sent to me by Dr. Ray Grayson, of Washington, Pa. It is a congenital malformation of the rectum. The rectum ends at the base of the bladder. It is interesting on account of the fact that we very seldom get a *post-mortem* in cases of this kind, and it represents a type of cases not at all uncommon. An examination of the rectum here discloses the fact that there is peritoneum connecting the rectum with the bladder. The rectum enters directly at the base. Some times the rectum enters the bladder at the vertex. After an examination is made, it will be seen that the peritoneum surrounds the entire lower part of the rectum running from the bladder directly to the rectum and surrounding it. This case represents one of a type of these cases of congenital malformation of the rectum which vary from occlusion of the anus to complete absence of the lower bowel. It is said that congenital malformations of the rectum and anus occur about once in every 5,000 deliveries, although some observers state that in statistics of 66,000 cases of delivery, congenital malformation of the rectum and anus occurred only three times. Other observers, however, state that congenital malformations do occur as often as one in every 5,000. To my knowledge, quite a number of cases have occurred around here. It is obvious from the malformation here that operative procedures were hazardous. An attempt in this case was made to reach the rectum but failed. The diagnosis was properly made of entrance of the rectum into the bladder by the appearance of the fæces in the urine. The operation, I believe, was made on the patient on the 17th day, and the patient lived until the 26th day.

DR. STEVENSON: I have seen three cases of imperforate anus; in one of the cases the rectum terminated in the bladder. In that case there was an attempt made to reach the rectum, but it failed and the child died. In two other cases I have seen the rectum was reached, and the method pursued was passing up a hypodermic needle and withdrawing the fæces and cutting up alongside of the needle, and the rectum was reached and drawn down and the opening stitched. These children both recovered, and had no trouble with their bowels.

DR. STEWART: It seems to me that in this case a suprapubic cystotomy would have been proper, and would have given relief.

DR. BUCHANAN: I think a very much better way would have been to open the sigmoid flexure of the colon; that can always be reached. It would be very much better to drain the fæces out by an abdominal fistula than through the bladder.

DR. McKENNAN: I find that operations in cases of this kind have never been successful. Operations have been done, some operators opening the perineum, cutting into the bladder and thence making a cut clear through the opening of the rectum into the bladder, making thus a large wound into the perineum. But this method of procedure either produces peritonitis or it causes a fistulous opening in the perineum, which greatly contracts. The only operation which can be done with safety is that suggested by Dr. Buchanan, and that is the operation of colotomy. I find that in malformations of the anus and rectum, that in which the rectum enters the bladder occurs in about forty per cent. of all malformations.

GENERAL DISCUSSION ON SURGICAL JOINTS.

DR. MURDOCH: I am not exactly clear as to what is meant by surgical joints. I suppose it may be joints liable to disease or injury, or that might come under the care of the surgeon, but in that case it would properly include every joint in the body, for there is no joint that might not require surgical treatment; therefore I do not like the term wholly. I suppose, however, reference is intended to be made to those joints which more frequently come under the care of the surgeon, either for disease or injury, and as that would be so much as to include the whole subject of tuberculosis and all kinds of injuries to the joints, I am not able or willing, and if I were there would not be sufficient time, to discuss the subject as a whole. It might be said, however, that there have been great changes in the surgical treatment of joints within a comparatively few years, as you are all well aware. This has arisen in a great measure from the fact that because of the great improvements in surgery since the introduction of antiseptic treatment of wounds the joint can be invaded and dealt with with so much less risk than formerly. That is one reason. And it seems to be a sufficient reason in the minds of a great many surgeons, that simply because joints can be gotten into and incised or scraped out, that is a good reason for doing it, and of course this must enter into the problem of whether such an operation should be done. Another reason why the joints are more frequently treated surgically now than formerly is owing to the changed views with regard to the chief disease which attacks the joint, namely, tuberculosis. Without entering into a discussion of the pathology of that diseases, we are all, I believe, convinced that the former ideas with regard to it were not correct. I think we all believe now that it is an infectious disease and is not always inherited from the parent. We believe the trouble is usually of local origin, and there is a local focus from which the disease starts, and it is in that view, I think, that a great many operations are now done by surgeons who would have formerly looked with doubt upon the idea that the local focus of the tubercle can be taken away before it has found localities in other parts of the body. In my recent visit to Europe, both in Ireland and Scotland I saw surgeons there opening into the joints in cases where I am sure nobody here in the United States would think of operating upon, nor do I believe they would be permitted to operate. I saw the joints of young people opened where there were none of the aggravated signs which we look for here, with a view of excising this local focus which it was believed existed either in the bone or in the joint. I saw, for instance, a surgeon, Dr. McEwen, of Glasgow, operate on a child about fourteen years old, able to walk without much limping, but afflicted with what we call the first stage of hip joint disease. I saw him cut into the joint and remove the head of the femur. In Ireland I saw a surgeon operating by what they call there an anterior procedure. In these operations they did it in the first stage of disease, before the disease had extended and made much or any destruction of the joints, but they do this operation on an entirely different principle from what I have been in the habit of seeing. They do it with the least possible violence to the joint; the head of the bone is not thrown out of its position. In both of these operations, Dr. McEwen did his operation posteriorly, making the usual incision from the crest of the ilium down from the joint, a short incision, and then introduced his chisel through an opening not over an inch and a half long, and by its manipulation, much pressure and lateral motion, he was able in a very short time to cut off the head of the bone, and then introduce his finger and extract the head. As I said before, I do not believe this would be permitted in our country. We see so little of joint diseases here, tubercular diseases, compared with what I saw in Ireland and Scotland. This is accounted for by the fact that the patients are not so well fed there. Among the poor in Scotland, the number of young people with joint disease is remarkable. Now, as I said before, I do not expect to be able to treat all of this subject, and I must say that I have had very little experience in the treatment of any of the joints, excepting that of the knee. I have had some experience in that, and have excised the knee some eight times, I think, and with seven successful cases. My friend, Dr. King, at the West Penn Hospital, has perhaps excised more, and has lost but one patient. I wish to speak of the difference between present practice and that in vogue when I was a young surgeon. I know of no subject which shows the great improvements that have been made in surgery more than this one of the manner in which the joints can be opened. During our late war, for gunshot injuries of the knee joint there were fifty-seven operations performed, and of these fifty-seven, forty-four patients died. Mr. Otis, in his report of our late war, states that previous to the war there were some eighteen excisions of the knee joint, of which sixteen were fatal. Now, the operation of excision of the knee joint is one that is almost universally successful, that is, the patient seldom dies under the operation, and it usually results in a useful limb. In Ireland, where they do this operation a great many times, with great success, I was shown at the Richmond Hospital some twelve cases that Dr. Thompson had in the hospital under recovery. He told me he had done the operation forty times, with only one death, so that no doubt the operation is one recognized as proper, when formerly amputation would have been in all these cases considered the proper course.

When I look back upon my practice, even as late as when I became surgeon of the West Penn Hospital, within twenty years, I can remember patients who lay there for a year or two years with white swelling, as we called it, and eventually perished. I have seen some of these cases amputated, and I have seen several of them succumb simply from the confinement and the inability of the doctors to do them any good. Now these cases would not be permitted to stay there two weeks before some surgical operation would be performed for their relief. As you know, a local focus exists in tuberculous disease; it may be necessary to incise the joint, but in other cases, when only the synovial membrane is involved, the operation of arthrotomy may be performed; opening the joint up widely and dissecting out the entire synovial membrane and scooping out with a gouge any local focus that may be found. The disease I do not believe ever commences in the cartilage. I desire, however, to state at this time, and it is probably all that is necessary for me to say to you to show the method that Dr. Thompson uses to the knee joint, after having opened it, that this is much superior to anything that I have seen, although it is a good deal like the apparatus which I use myself.

I have brought it with me and will show it to you. In operating on a knee joint, they are in the habit of making what is called the horseshoe incision. This is made by commencing well back, and carrying the knife downwards and upwards across to a corresponding point on the opposite side, the joint opened, and if it is only desired to perform arthrotomy, the whole of the membrane is scraped with a scoop and cut away with the scissors, and then the flap is replaced. But if, on the other hand, it is desired to perform excision of the joint, the bones are cut off and fastened together with nails and a splint. The design of those who operate by cutting parallel with the articular surface is to leave the limb at the same relative angle. Dr. Thompson and those surgeons who have had the most experience in operating tell me that it is not the proper way to make the section of the femur; he makes the section of the femur at right angles with its axis, so as to make the leg perfectly straight, as it is in the normal leg. I am inclined to believe that is the better way. I will not go into the manner of cutting the bone, as the surgeons all know that as well as I do. The best way of fixing the limb, that is the important part of the operation. I presume part of the success of this operation in recent years had been owing to this fact. Older surgeons had been in the habit of using wire and other appliances, which did not accomplish the purpose very well. I believe the idea of doing anything to keep the parts in apposition originated in Germany, by the use of steel nails driven with a mallet into the bones. I do not think that was as good a means for keeping the bones in place as the one suggested by me. In Ireland they use silver pegs about an inch and a half long, after making a hole with the brad awl. The nails which I use are four and a half inches long for an adult. They are made for me by Mr. Helmold, and according to the pattern of Mr. Wyeth. The nail should be tapered so that it binds as it proceeds. Three nails should be used. Then hold the bones in perfect apposition with the assistance of the external apparatus. The apparatus which Dr. Thompson uses, and which I think is the best way to hold the limb steady, is made from common hoop iron, an inch and a half wide. This is easily manipulated; it is simply wrapped around with a bandage over it to hold it in place, an anterior and posterior splint. The posterior splint is put down around the ankle joint and up on the foot, the anterior one leaving a space for the dressing over the knee joint, and after the operation it is not disturbed for three weeks, unless the elevation of temperature is over 100° F. There is a drainage tube put in across the joint behind the bone, well down, and usually it is a very successful operation. I could relate some of my cases, but I will not trouble you with that; the time is passing. I will however mention a case that I operated upon at the West Penn Hospital, a man 47 years old, a miner, suffering with disease of the joint. Although in this case I feared the operation could not be very successful, the man made a remarkable recovery. He walked into the operating room four weeks after the operation with a cane, and left the hospital in eight weeks. He had been suffering for two or three years. I received a letter from him three months after he left the hospital. He said: "With the greatest of pleasure I let you know that I am walking without crutch or cane. It was on the 5th of February that I walked. I was very much surprised at myself when I did it. From the day that you operated on my knee until the day that I walked was four months and eighteen days. How is that for an old man? Therefore, I thank you most respectfully for your skillful operation on me."

The joints in which operations are the most useful and in which the surgeons now have the most experience and have done the most benefit are the knee, the hip and the elbow. Excision of the elbow for injury is a most successful operation; so is excision of the knee. But I will say, as I said in the beginning, that there are many surgeons who think that because excision of the joint is done with such safety there is a good reason for doing it. It should always be remembered, especially by the young surgeon, that an excised joint is an admission on the part of the surgeon that he is not able to cure it. As our knowledge of tuberculosis advances, and we are able to treat tuberculosis successfully in the lung, we will be able to treat it successfully in the joint, and operative interference will not probably be essential then. It should never be forgotten, as the very first principle in the treatment of all joints, that the first consideration is rest, putting the parts at rest. If joints can be kept still even where there is a local focus of tuberculosis, if they can be kept still, and proper hygienic measures resorted to, many cases will never call for aid from the surgeon. I believe the improvement in the treatment of diseases rests in an early diagnosis, and early treatment. Having said this much with little regard for order, I leave the matter in your hands.

DR. DAVIS: The term surgical joints has been used to describe joints that call for surgical interference.

DR. STEVENSON: I have never made claims to being a surgeon, but I have been so situated that I have had to do a little surgical work. I practiced for twelve years in Westmoreland county; I was medical man, surgical man, obstetrical man, and so forth. I had charge at that time of the Penn Gas Coal Company's works, which employed some seven hundred men, and I necessarily saw a good deal of injury. I think the first case I saw after I opened the office was a compound fracture of the ankle joint, with dislocation of the tibia. After cutting and having two or three men exert all the strength they could, I could not get the tibia returned into the joint, so I found a meat saw and sliced off about half inch and got it reduced, and that man is walking about to-day. I saw not long after that a carpenter doing something with a foot adz, the corner of the adz striking him just over the joint, and penetrating the joint. When I saw him the synovial fluid was exuding. This being before the era of antisepsis it ended in an amputation about four inches above the knee joint. The man got well with the loss of the limb. I have no doubt the improved methods of treatment would have saved that man's leg. I saw another case which was probably a tuberculous joint. It seemed to start without any known cause, and after continuing quite a number of months, the joint suppurated and I found it necessary to amputate above the knee. That man was not so fortunate as the other, his general health gave way and he died, although the stump had healed and done fairly well. One of the first important things is the diagnosis. What have we? Now in joints, we have a great many structures, there is bone, there is cartilage, there is synovial membrane and ligaments and the surroundings. Any or all of these may be involved, or none of them may be. We have what is called simulated disease in joints the same as we have simulated diseases of other organs. We may have a mimicry of disease in a joint, and this may simulate almost anything. It is a very important matter when a surgeon or practitioner is called to a lady, nervous, of inherited tendencies, want of stability, easily excited mentally, and finds that she is complaining of severe pain in her knee. You look at the joint, you see it is swelled; she says she cannot use it, you attempt to use it, she screams out with pain. No doubt it is very important to determine whether it is a hysterical joint.

The constitutional history of the patient may decide this, but if you have an inflammation of the knee joint, you will have local heat. Possibly, you will have constitutional heat. If you feel this joint and it is cool or clammy, and you take the temperature of the patient, and you find there is no fever, there is strong ground for suspecting that you have no chronic trouble in the knee joint.

Dr. Batten: In speaking of operations for joint diseases I will not go into a discussion upon surgical treatment. I believe it has been established that these diseases are of a scrofulous nature, and it was believed that that was a fact up to the time that Koch discovered his bacilli. Since that it is believed that the tubercle bacilli caused all these conditions of the joint, and that they are not hereditary. There is a question in my mind whether they are not hereditary. I believe the bacilli can be carried from the mother, a phthisical mother or a scrofulous mother, to the infant. However, that is a question. But there is one case I know in which an operation was not performed. It was a boy about ten years old, whose parents were living. He had what was called white swelling or inflammation of the knee joint. He was placed under the care of a great many physicians or surgeons, but there were no operations performed, and he finally recovered from this condition, and is at the present time using all the joints and is an active, healthy man. I would say, however, that Dr. Murdoch is deserving of a great deal of credit for the manner in which he performs these operations, and the success that he has had in giving relief to the patients upon whom he operates.

DR. KŒNIG: In surgery, I think we all admit, cleanliness ranks superior to godliness. In view of the recommendations that Dr. Murdoch has made of a certain instrument—the little household utensil with which he inserts his nails—it seems to me that we must accord him greater godliness than cleanliness. With his well known ingenuity he should be able to construct some appliance capable of being made aseptic, after which he would have no occasion to recommend the use of an instrument as crude as the one he has shown us.

DR. LANGE: I have recently seen a few surgical points. I will relate one or two cases. A boy about eight years old while playing on the carpet screamed, said he had hurt his knee, and when his mother got it uncovered, she found on the most prominent part of the knee a single drop of blood, which was wiped away and the little fellow moved around the house, but limped. His mother instituted a search for needles and found half a needle with the thread in its eye. The accident did not seem to trouble the little fellow much until the third day. Although there was no swelling and very little heat, there was a good deal of pain, and when called, I considered it probable a piece of the needle was in the joint or about the joint, and that it would be the proper thing to anæsthetize the boy and attempt to remove it. This was done, a careful search was made for the piece of needle for more than an hour and a half. The joint, however, was not entered. After that, the little fellow was put to bed and his limb on a straight wooden splint; he was kept in that way two weeks and then allowed to get up. He was up about a week and was again seized with pain and this time a distinct fullness of the joint. The four depressions at the four corners of the patella had disappeared and were replaced by four convexities which fluctuated.

The leg was put in plaster and all motion of the knee joint was prohibited by the plaster for three months. Then the plaster was taken off and the boy beginning to be active, there was again a slight swelling of the joint, and the plaster was reapplied and kept on for a couple of months more, and then taken off; and finally we saw the end of that surgieal joint. The needle has, in all likelihood, become encysted, and will likely do no more harm. The other case was that of a boy riding his velocipede and falling with it. He was picked up and carried home, and when his doctor saw him he concluded he had a dislocation of the femur, because the leg was fully an inch and a half or two inches longer than the other, and because it was rigid, immovable and painful. The doctor chloroformed him, and attempted to reduce the dislocation, and thought he had succeeded. He applied a bandage to the boy's thigh and pelvis, and put him to bed, and the boy complained very little for two or three days. After this the doctor took off his bandages, examined the limb, and found it was fully two inches longer than the other. It was then I saw the boy and examined him under chloroform as the doctor had done. The curious part of the case was that when the boy was anæsthetized his limb was the same length as the other, and it was evidently not dislocated; but when the boy came from under the influence of the anæsthetic, the limb lengthened two inches. The parents sent for additional counsel, and the last medical gentleman called in concluded that the boy had hip joint disease. We could not make a diagnosis, allowed that to go, and put the boy to bed.

He was kept there two or three days, then got up and walked, and had no pain or deformity. On a later occasion when I saw him he complained of pain, and again his leg was apparently two inches longer. We examined him very carefully, and we found that this was a simulated disease, that, as my friend, Dr. Stevenson, has characterized it, it was an hysterical joint, and that the lengthening was not between the pelvis and the femur, but was produced by muscular tilting of the pelvis. The length from both anterior superior spinous processes, to corresponding points below, was always the same, even when the leg projected two inches beyond its fellow. On the other hand, a line from one anterior superior spinous process to the other is not at right angles with the body, but two inches lower, on the side where the leg seems longer. This boy is now actively about, painless and straight; but when he is cross, willful, or disappointed, he complains of his hip, tilts his pelvis, and lengthens his leg.

DR. GREEN: Dr. Lange's case reminds me of a surgical joint with which I have had some trouble. The patient, whom I have been called to see many times, has the power of dislocating the lower jaw. She is a girl of nine years; she has always been notorious for will power. Her mother told me that from childhood, whatever she asked for had to be given her. She would say: "If you don't give it to me I'll stretch," and immediately, were the request not granted, the child would begin to "stretch," and open her mouth just as wide as she possibly could, until her jaw would slip out. About two months ago I replaced the jaw; whether she has done much stretching since that time I do not know. I have known some persons who frequently had dislocations of the lower jaw, but in no other case have I seen a person who could willfully, maliciously, bring about this condition of affairs by stretching, and this boy of whom Dr. Lange has spoken reminded me of the spoiled child who "stretches."

DR. BUCHANAN: I understand the subject of the evening to be surgical joints, and those, I presume, are joints which are subjects for surgical treatment, either from disease or accidental injury. Vast improvements have been made in the treatment of injured joints within the last ten or fifteen years. It is within my recollection when a simple puncture of the ankle joint, and an injury requiring amputation of the anterior part of the foot, would have determined a Syme's operation, or an amputation of the leg. So great stress was laid upon the fact that *a joint had been opened*, and I believe this to be true with very many medical men to-day, that, when called to such a case, the question of amputation rises strongly in their minds. It is well known to-day by surgeons that the synovial membranes can be treated in very much the same way, and with the same impunity as the tendinous sheaths, or any other of the soft tissues of the body. The thing of importance is, when these cavities are open, to keep them aseptic. If this is done no harm can result from the opening, and in the case of the joints we have exactly the same means of keeping them aseptic as in the case of the peritoneal cavity, and we can, in addition, if desired, use antiseptic solutions. Now, we are constantly called to dress injuries of joints, particularly fractures of the bones which go to form the joints; and I am satisfied that the practice will be in the future, in many cases, to open joints, wash them out, repair the soft parts, and wire the fragments of bone where the joint has been subcutaneously opened, and where the bones cannot be kept in apposition without great trouble, painfully pressing splints and firm bandaging. I am reminded of a case which Dr. Murdoch saw with me in consultation about a year ago. The patient had a simple fracture of the fibula and a fracture of the inner malleolus. I was called to the case and reduced the fracture without great difficulty, and was able to place the broken malleolus exactly in its position, and retain it there with a simple splint. Dr. Murdoch was called in consultation at the request of the patient, and, to my satisfaction, the next day. We endeavored to replace this dressing by another more permanent in character. This set up a frightful spasm of the muscles of the fibular side of the limb, and the spasms were so great that, using all our force, we had not the power to overcome them and place the limb in shape. I never saw a patient suffer more than did this patient for a few minutes. These sharp-edged fragments threatened to break through the skin and form a compound fracture. I proposed at that time, although the patient refused to listen to any suggestion of the kind, to make an incision over the point of fracture, and put in a single silver wire to retain the inner malleolus in position, and the pressure of an ounce or two ounces on the silver wire would perfectly keep the bone in position. Having to start with a simple fracture, having made the wound ourselves, we could keep it aseptic, no harm could come to the joint. I believe the time will come when that will be the ordinary treatment for such fractures in the neighborhood of joints, where the disposition to displacement is very great, where a very slight force exerted through a silver wire will hold the parts perfectly in apposition, and where we have every possible chance to keep the wound aseptic. There is another aspect of surgical joints not dealt with very often, and that is the advisability, where there is doubt, of making an exploratory opening. I see no reason in the world why exploratory openings should not be made into joints when we suspect disease, as well as into the peritoneal cavity, and as often; but such openings are, I believe, very rare. With regard to cases reported this evening, of compound fracture of the inner malleluos and fibula, in which there was protrusion of the shaft of the tibia, in which the patient was etherized, and section of the tendo. Achillis made, and a piece cut from the end of the tibia to facilitate reduction, I would say that I reported to this society a year ago a case exactly similar in all respects. I did not find it necessary to do a tenotomy, and the bone was returned without sawing any of it off, and I can hardly imagine a case of this nature in which the same result could not be secured, providing the opening in the soft part is sufficiently large to let the bones slip in. Muscular action is the only thing that would prevent the return of such a bone, and it can be completely abolished by anæsthetics.

DR. DAVIS: All cases of joint diseases present certain characteristics peculiar to themselves, and require good judgment on the part of the surgeon at the time, and can scarcely be discussed in a general way, but there are joints that are difficult of diagnosis, that no doubt give rise to a great deal of distress to the patient and give rise also to a good deal of distress to the attending physician, because of the long continued suffering involved. The youngest practitioner is likely to come in contact with such joints. One of the very important questions in such joints is when a surgical operation is advisable, or whether it is advisable at all. Take, for instance, the tuberculous joints referred to. The question comes up, when to operate upon it. Will the opening of these joints remove the diseased tissue? Will it give the patient a better chance of life? It is but a few years since all cases of hip joint disease were considered the property of the surgeon. We have heard to-night of this being carried to extremes on the other side of the water, and operations done which would not be allowed here, and yet in looking at the statistics this operation on the hip-joint has not been satisfactory. In the first place, quite a large percentage of those operated upon have died ; perhaps not directly following the operation, but within a few days or a few weeks after it. And of those who have recovered from the direct result of the operations, over one-half have died where the diseases have been of tuberculous origin, in such a short time that it is questionable whether the operation does not hurry the general disease. I have read somewhere that out of 388 cases of hip joint disease operated on, only sixty-one presented results that could be called satisfactory. Of these sixty-one there were about forty that had motion in the joint. Of the forty there were about ten who did not have to use artificial means, such as crutch or cane in walking. Results such as these are not flattering for the operation, and do not lead us to hurry or advise our patient to go into the hands of the surgeon and submit to the operation so liable not to be favorable in its outcome. And then in regard to operations on the knee. While we know that under aseptics it has improved wonderfully as regards immediate death, yet the cases especially where it is tuberculous have not done as well as we could wish. And so of the ankle joint. I have in mind now a tuberculous ankle joint where operations have been advised over and over again. I do not know but that if this young man would submit to the operation, and have all this bone removed, the confinement in the house would hasten his end. It is difficult for me to know whether to advise an operation or not. The difficulty with the general practitioner is to know whether to turn such cases over to the surgeon, and with the surgeon to know whether operation ought to be resorted to.

DR. MURDOCH : I think the surgeons who are in advance in this matter of treatment of joints are tending toward diminution of operations at the late stage, where there is great injury to bone. Patients in that condition do not recover well from an excision of a joint, and I believe the tendency of the better surgeons now-a-days, would be to recommend radical measures in joints such as Dr. Davis has described. If the bones are extensively diseased, and the joint extensively involved, the patient would be likely with an excision, to perish from a general giving away of the system. I fully believe that such surgeons as McEwen, of Glasgow, and Barker, of London, and the surgeons most in advance, operate early, when the disease is local, and before it has yet attacked the synovial membrane, then is the time to operate at the earliest stage, when it is possible to remove the disease, and it can then be removed through a smaller incision without disturbing the relations of the joints, and a movable articulation may then be possible. In Ireland and Scotland, where there are hundreds of these cases to one here, the people have been educated up to the necessity of not allowing this disease to go on, and are willing to submit to an early operation. I am very sure the people here will not submit to the early operation thought proper there. I believe that it will yet come to be the proper practice where a diagnosis can be made sufficiently early. I will say to Dr. Kœnig that if he will come up some time to my operations, I will show him how to do the operation, and how I have been so successful in preventing infection of the wound with the washouts I use.

